# Expression and significance of transforming growth factor-β1 in epithelial ovarian cancer and its extracellular matrix

**DOI:** 10.3892/ol.2014.2448

**Published:** 2014-08-14

**Authors:** KEMING CHEN, HUA WEI, SHENGRONG LING, CUNJIAN YI

**Affiliations:** Department of Gynecology, First Affiliated Hospital of Yangtze University, Jingzhou, Hubei 434000, P.R. China

**Keywords:** transforming growth factor-β1, ovarian cancer, extracellular matrix

## Abstract

The aim of the present study was to investigate the expression and significance of transforming growth factor-β1 (TGF-β1) in the cytoplasm and extracellular matrix (ECM) of epithelial ovarian cancer cells. The expression of TGF-β1 protein was detected in paraffin-embedded sections of 25 normal ovarian epithelial tissues, 10 benign epithelial cysts and 72 epithelial ovarian cancer specimens, using the Strept Avidin Biotin Peroxidase Complex immunohistochemistry method. In addition, the expression of TGF-β1 mRNA in normal fibroblasts (NFs) and ovarian cancer-associated fibroblasts (CAFs) was assessed using semi-quantitative polymerase chain reaction (PCR). TGF-β1 protein was expressed in the cytoplasm and ECM, and no significant difference was identified between normal and benign ovarian tissues (P>0.05). However, the cytosolic expression of TGF-β1 declined gradually between the benign ovarian tumor and epithelial ovarian cancer, while its expression in the ECM significantly increased (P<0.05). The expression of TGF-β1 in the cytoplasm and ECM in epithelial ovarian cancer was found to negatively correlate with tumor differentiation, however, it was positively associated with the clinical stages. The positive rates of TGF-β1 in the cytoplasm and ECM between ovarian cancers in clinical stages I–II and III–IV were significantly different (P<0.05). Furthermore, the PCR data indicated that the relative expression of TGF-β1 mRNA in ovarian CAFs (1.0270±0.0539) was significantly higher than that in NFs (0.7131±0.0186). Therefore, the expression of TGF-β1 was identified to be associated with the development and progression of epithelial ovarian cancer, and the high expression of TGF-β1 in the ECM may be associated with the invasion and metastasis of ovarian cancer.

## Introduction

Tumor formation is a complex process. The initiation and development of a tumor involves multiple signaling pathways, including various aspects of cell proliferation, inhibition of apoptosis, tumor stroma and formation of peritumor blood vessels, which are all regulated by a variety of factors. Recently, the effect of cytokines on tumors has been the focus of attention. Transforming growth factors (TGFs), including TGF-α and -β, are important factors that regulate cell growth and differentiation (1).

TGF-β1 inhibits tumor cell proliferation, however, it also promotes tumor growth and invasion by modulating the tumor microenvironment, promoting the formation of tumor blood vessels and matrix, and suppressing the immune response. Therefore, the present study detected the expression of TGF-β1 in the cytoplasm and extracellular matrix (ECM) of epithelial ovarian cancer cells, to investigate the association between its expression, and the invasion and metastasis of ovarian cancer.

## Materials and methods

### Materials

A total of 72 paraffin-embedded epithelial ovarian cancer samples, obtained from the First Affiliated Hospital of Yangtze University (Jingzhou, China) between March 2001 and 2006, were included in the present study (Table I). The mean patient age was 45.3 years. The 25 normal ovarian epithelial tissues used in the study were collected from patients with uterine disorders, who had received a hysterectomy with concurrent removal of the ovaries, or from a normal ovarian tissue biopsy; the mean patient age in this group was 43.5 years. Ten random samples of benign ovarian cysts (serous [n=6], mucinous [n=3] and endometrial [n=1]) were also included and the mean age of the patients in this group was 41.7 years. Three lines of epithelial ovarian cancer-associated fibroblasts (CAFs) and three cultures of normal fibroblasts (NFs; derived from normal ovarian tissues resected due to early cervical cancers) were provided by the Laboratory of Gynecologic Oncology of Union Hospital (Wuhan, China). Ethical approval for the present study was obtained from the First Affiliated Hospital of Yangtze University Ethics committee.

### Immunohistochemistry

Immunohistochemical staining was performed using the Strept Avidin Biotin Peroxidase Complex kit purchased from Beijing Zhongshan Golden Bridge Biotechnology Co., Ltd. (Beijing, China). The primary polyclonal rabbit antibodies (1:100) were purchased from Santa Cruz Biotechnology, Inc. (Santa Cruz, CA, USA) and staining was performed according to the manufacturer’s instructions.

### Semi-quantitative polymerase chain reaction (PCR)

The relative expression of TGF-β1 mRNA was detected using total RNAs extracted from the epithelial ovarian CAFs and NFs using TRIzol reagent (ShineGene, Shanghai, China). Total RNAs were reverse-transcribed to cDNA; 1 μl was used for TGF-β1 amplification. PCR conditions were as follows: Pre-denaturation, 94°C for 4 min; denaturation, 94°C for 45 sec; annealing, 55°C for 1 min; extension, 72°C for 1 min for 29 cycles. Final extension was at 72°C for 5 min. The specific primers used for the TGF-β1 gene (199 bp product) amplification were as follows: Forward, 5′-CAACAATTCCTGGCGATACA-3′ and reverse, 5′-GGTAGTGAACCCGTTGATGTCC-3′. The β-actin gene (619 bp product) was used as an internal reference and the primers used were as follows: Forward, 5′-AAGAGAGGCATCCTCACCCT-3′ and reverse, 5′-GGAAGGAAGGCTGGAAG-3′. Following the reaction, PCR products were separated on 2% agarose gel by electrophoresis.

### Immunohistochemistry

According to the staining results, cytoplasm or ECM exhibiting brown granules were considered positive for TGF-β1. Based on the pigmentation intensity; no pigmentation, light yellow, yellow or brown, the positivity was scored as 0, 1, 2, or 3, respectively. Five different regions of each section were selected to calculate the average percentage of positive cells and the corresponding scores. Sections with <5% positive cells scored 0; 5–25% positive cells scored 1; 26–50% positive cells scored 2; 51–75% positive cells scored 3; and >75% positive cells scored 4. The two scores were then multiplied to determine the positivity of a sample, whereby 0–2 corresponds to (−), 3–4 to (+), 5–8 to (++) and 9–12 to (+++) (2). The results were examined by two individuals to reduce error and bias. For semi-quantitative PCR analysis the gel electrophoresis results were recorded using the Gel Doc XR System [Bio-Rad Laboratories (Canada) Ltd., Mississauga, ON, Canada] and the densitometry was analyzed using an automatic image analysis system (Gel Doc XR System) to calculate the relative contents of TGF-β1 mRNA (β-actin served as the internal reference). The following formula was used: TGF-β1 mRNA relative expression level = TGF-β1 band density/β-actin band density.

### Statistical analysis

Statistical analyses were performed using SPSS version 13.0 (SPSS, Inc., Chicago, IL, USA). The immunohistochemistry results were analyzed using a non-parametric test and the results of semi-quantitative PCR are presented as the mean ± standard deviation. The data were compared by t-test and P<0.05 was considered to indicate a statistically significant difference.

## Results

### Expression of TGF-β1 in epithelial ovarian tumors

**Figure 1:**
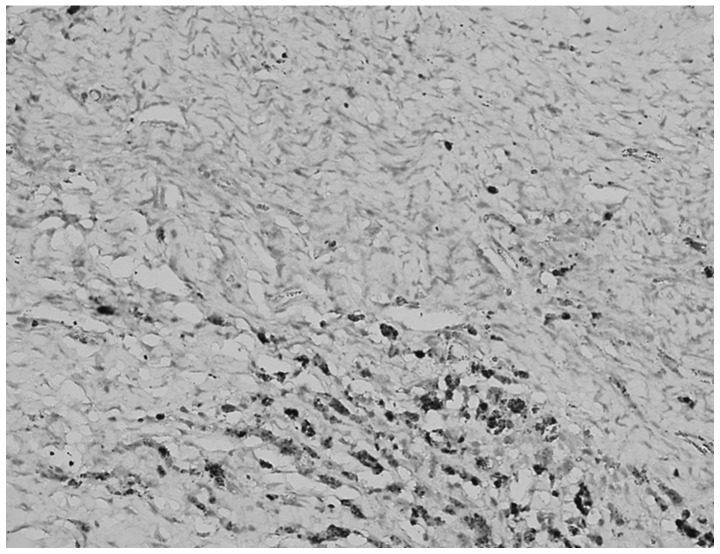
Expression of transforming growth factor-β1 was (+++) in the cytoplasm and (+) in the extracellular matrix of normal ovarian epithelial cells (magnification, ×200). Sections with <5% positive cells scored 0; 5–25% positive cells scored 1; 26–50% positive cells scored 2; 51–75% positive cells scored 3; and >75% positive cells scored 4. To dermine the positivity of a sample 0–2 corresponds to (−), 3–4 to (+), 5–8 to (++) and 9–12 to (+++).

**Figure 2:**
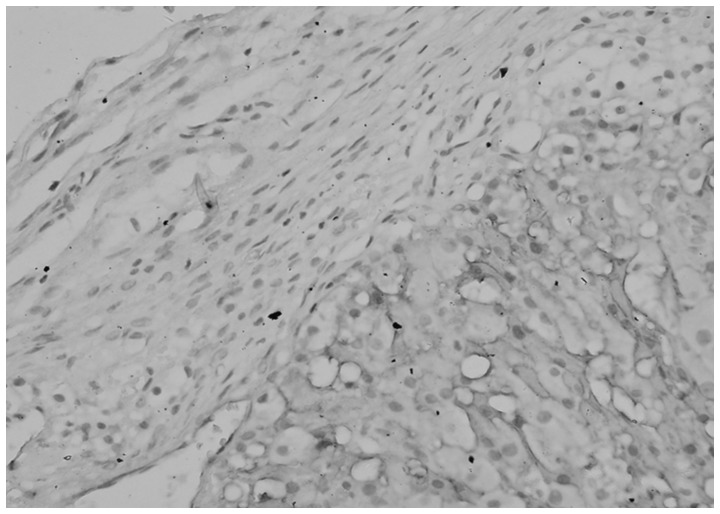
Expression of transforming growth factor-β1 was (++) in the cytoplasm and (−) in the extracellular matrix of benign ovarian epithelial cells (magnification, ×200). Sections with <5% positive cells scored 0; 5–25% positive cells scored 1; 26–50% positive cells scored 2; 51–75% positive cells scored 3; and >75% positive cells scored 4. To dermine the positivity of a sample 0–2 corresponds to (−), 3–4 to (+), 5–8 to (++) and 9–12 to (+++).

No significant differences were identified between the expression of TGF-β1 in the cytoplasm and ECM of normal ovarian tissue and benign tumors (P>0.05), however, the levels of TGF-β1 in the cytoplasm and ECM were significantly different between epithelial ovarian cancer and the corresponding normal ovarian tissue (P<0.05; Table II, Figs. 1 and 2).

### Association between TGF-β1 expression in epithelial ovarian cancer, and clinical stage and pathological grade

**Figure 3:**
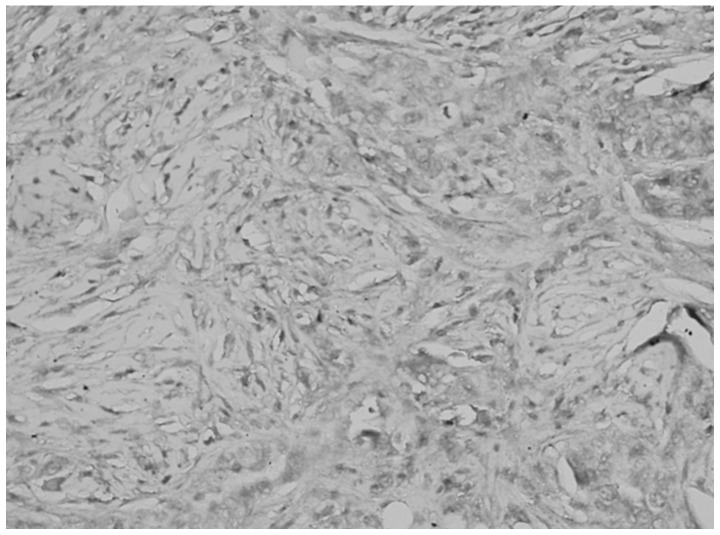
Expression of transforming growth factor-β1 was (++) in the cytoplasm and (+) in the extracellular matrix of epithelial ovarian cancer cells (magnification, ×200). Sections with <5% positive cells scored 0; 5–25% positive cells scored 1; 26–50% positive cells scored 2; 51–75% positive cells scored 3; and >75% positive cells scored 4. To dermine the positivity of a sample 0–2 corresponds to (−), 3–4 to (+), 5–8 to (++) and 9–12 to (+++).

**Figure 4:**
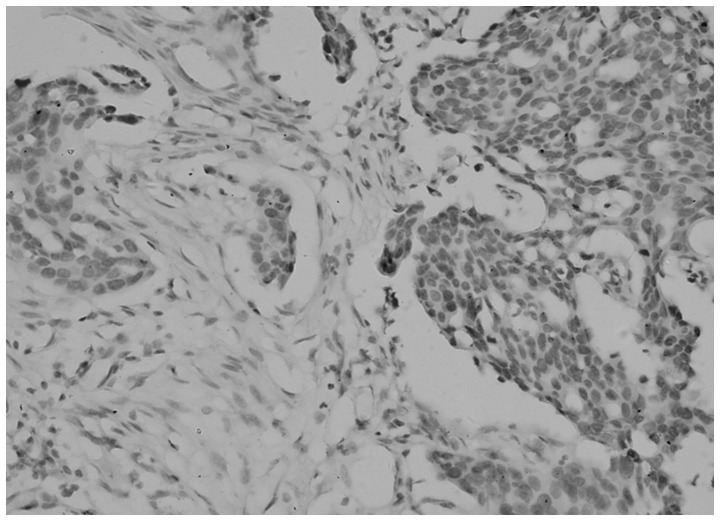
Expression of transforming growth factor-β1 was (−) in the cytoplasm and (−) in the extracellular matrix of epithelial ovarian cancer cells (magnification, ×200). Sections with <5% positive cells scored 0; 5–25% positive cells scored 1; 26–50% positive cells scored 2; 51–75% positive cells scored 3; and >75% positive cells scored 4. To dermine the positivity of a sample 0–2 corresponds to (−), 3–4 to (+), 5–8 to (++) and 9–12 to (+++).

The differences in TGF-β1 expression in the cytoplasm and ECM were identified as statistically significant between the pathological grades and stages (P<0.05; Table III, Figs. 3 and 4).

### Detection of TGF-β1 mRNA levels using semi-quantitative PCR

**Figure 5:**
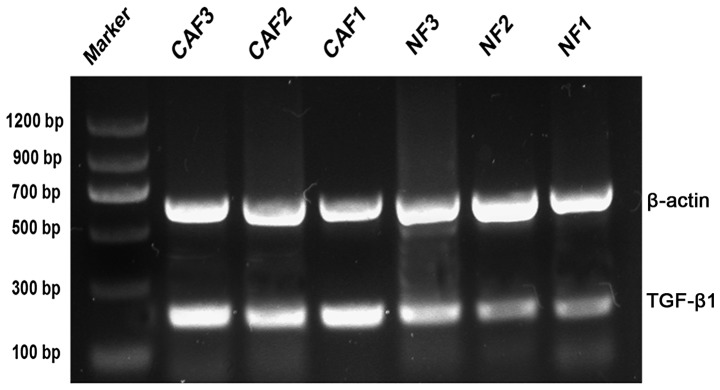
Electrophoresis detection of the polymerase chain reaction products of TGF-β1 mRNA in ovarian CAFs and NFs. β-actin served as an internal reference. CAF, cancer-associated fibroblast; NF, normal fibroblast; TGF-β1, transforming growth factor.

The relative levels of TGF-β1 mRNA in CAFs and NFs were 1.0271±0.053 and 0.7131±0.0186, respectively. The levels of TGF-β1 in CAF1, 2 and 3 were 1.1271±0.0642, 0.9886±0.01130 and 0.9654±0.0863, respectively, whereas those in NF1, 2 and 3 were 0.6334±0.0188, 0.608±0.0060 and 0.8980±0.0309, respectively. The TGF-β1 mRNA level was significantly upregulated in CAFs compared with that in NFs (P<0.05; Fig. 5).

## Discussion

The inhibitory effect of TGF-β1 on the growth of normal and early stage tumor cells is achieved via inhibition of the cell cycle progression from the G1 to the S phase. Alexandrow and Mose (3) reported that the treatment of numerous cell lines with TGF-β1 led to a rapid decrease in c-myc mRNA and protein levels, while overexpression of the c-myc protein antagonized the inhibitory effect of TGF-β1 on cells entering the S phase. These results indicate that TGF-β1 regulates c-myc expression at the transcriptional and post-transcriptional levels, thereby inhibiting the c-myc-regulated cell functions and arresting cell growth in the G1 phase. The results of the present study indicated that the expression level of TGF-β1 was relatively high in normal ovarian tissue and benign ovarian tumors, although the positive expression rate of TGF-β1 decreased gradually from benign ovarian tumors to epithelial ovarian cancer. Furthermore, the expression of TGF-β1 in advanced stage and poorly-differentiated epithelial ovarian cancer was significantly lower than that in early stage and well-differentiated tumors, indicating that the autocrine loop of TGF-β1 may be involved in the process of apoptosis in tumor cells (4), which was suppressed in the advanced and poorly-differentiated tumors. The suppressed TGF-β1 signaling pathway and the subsequently weakened-inhibition of the c-myc proto-oncogene accelerated tumor cell proliferation and tumor progression.

Epithelial ovarian tumor tissues and the majority of ovarian cancers have a closely associated tumor stroma (5). The occurrence and development of cancer is not determined by epithelia or stroma alone, but by the equilibrium of the microenvironment at the tumor-host interface, which is formed as a result of the interaction between the two components (6). Among them the predominant host cells, CAFs, are important for regulating the equilibrium of this interface system. Numerous studies have indicated that during the process of tumorigenesis, the stroma surrounding epithelial cells is activated, which forms a cancer-associated stroma, subsequently promoting the occurrence and development of cancer (7). The predominant feature of an activated stroma is the transformation of fibroblasts into CAFs. *In vitro* and *in vivo* studies have demonstrated that TGF-β stimulates the conversion of fibroblasts into the phenotype of CAFs, indicating a critical role for TGF-β in the formation of a cancer-promoting stromal environment (8). Rosenthal *et al* (9) reported that TGF-β1 upregulates the expression of CAFs, while Xu *et al* (10) found that the TGF-β-treated SMMC-7721 hepatocellular carcinoma cell line altered significantly, adopting a spindle-shaped morphology, with reduced expression of E-cadherin and induction of β-catenin nuclear translocation, enhancing the cell motility. Previous studies have also shown that TGF-β1 promotes the expression of matrix metalloproteinase-2 (MMP-2) via the binding of transcription factors c-Jun and c-Fos to the AP1 (Jun/Fos) site in the MMP-2 gene promoter, thereby stimulating the release of MMP-2 from the tumor and surrounding stromal cells (11). MMP-2 degrades the intercellular matrix, as well as the major component of basement membrane, collagen IV, thereby hydrolyzing the basement membrane, which allows tumor cells to enter the connective tissue. TGF-β1 affects the ECM in a paracrine manner, exerting its effects to enhance the interaction between cancer cells and the ECM, which promotes angiogenesis and the suppression of the immune response, to provide a suitable microenvironment for cancer cells to accelerate their growth and metastasis.

In conclusion, the present study demonstrated that the ability of advanced epithelial ovarian cancer to produce autocrine TGF-β1 was declined or eliminated. This resulted in a weakened effect of TGF-β1 with regards to the inhibition of tumor proliferation and the promotion of tumor cell apoptosis, resulting in an overall reduction in its tumor suppression effect. However, in the stroma, the paracrine mechanism of TGF-β1 in cancer cells remained relatively normal and the released TGF-β1 exerted the abovementioned effects on the ECM. Recent studies have shown that the application of a TGF-β1 antibody, TGF-β1 binding protein or antisense oligos against TGF-β1 may neutralize the effect of TGF-β1, to achieve antitumor invasion and metastasis. Therefore, further studies regarding the association between TGF, and the initiation and development of ovarian cancer may provide novel insights into the diagnosis and treatment of the disease.

## Figures and Tables

**Table I:** Pathological grade and histological type of epithelial ovarian cancers.

Pathological grade	Serous type	Mucinous type	Endometrioid and clear cell types
G1	9	11	5
G2	10	7	3
G3	18	5	4
I–II	15	11	5
III–IV	23	11	7

**Table II:** Expression of transforming growth factor-β1 under various pathological conditions.

		Cytoplasm [n, (%)]				Extracellular matrix [n, (%)]			
			
Pathology	Cases	−	+	++	+++	−	+	++	+++
Normal ovarian tissue	25	6 (24)	5 (20)	8 (32)	6 (24)	21 (84)	2 (8)	2 (8)	0
Benign ovarian tumor	10	2 (20)	2 (20)	3 (30)	3 (30)	6 (60)	2 (20)	2 (20)	0
Epithelial ovarian cancer	72	42 (58)	12 (17)	10 (14)	8 (11)^a^	34 (47)	15 (21)	11 (15)	12

a P<0.01 vs. normal overian tissue.

Sections with <5% positive cells scored 0; 5–25% positive cells scored 1; 26–50% positive cells scored 2; 51–75% positive cells scored 3; and >75% positive cells scored 4. To dermine the positivity of a sample 0–2 corresponds to (−), 3–4 to (+), 5–8 to (++) and 9–12 to (+++).

**Table III:** Correlation between transforming growth factor-β1 expression, and the histological grade and clinical stage of epithelial ovarian cancers.

		Cytoplasm [n, (%)]					Extracellular matrix [n, (%)]				
			
Pathology	Cases	−	+	++	+++	P-value	−	+	++	+++	P-value
Histological grade						0.002					0.001
Well-differentiated	25	8 (32)	6 (24)	6 (24)	5 (20)		18 (72)	3 (12)	3 (12)	1 (4)	
Moderately-differentiated	20	12 (60)	4 (20)	2 (10)	2 (10)		10 (50)	4 (20)	4 (20)	2 (10)	
Poorly-differentiated	27	22 (82)	2 (7)	2 (7)	1 (4)		6 (22)	8 (30)	4 (15)	9 (33)	
Total	72	42 (58)	12 (17)	10 (14)	8 (11)		34 (47)	15 (21)	11 (15)	12 (17)	
Clinical stage						0.001					0.020
Stages I–II	31	11 (35)	8 (26)	6 (19)	6 (19)		21 (68)	5 (16)	3 (10)	2 (6)	
Stages III–IV	41	31 (76)	4 (10)	4 (10)	2 (5)		13 (32)	10 (24)	8 (20)	10 (24)	
Total	72	42 (58)	12 (17)	10 (14)	8 (11)		34 (47)	15 (21)	11 (15)	12 (17)	

Sections with <5% positive cells scored 0; 5–25% positive cells scored 1; 26–50% positive cells scored 2; 51–75% positive cells scored 3; and >75% positive cells scored 4. To dermine the positivity of a sample 0–2 corresponds to (−), 3–4 to (+), 5–8 to (++) and 9–12 to (+++).
